# Use of Chou’s 5-steps rule to predict the subcellular localization of gram-negative and gram-positive bacterial proteins by multi-label learning based on gene ontology annotation and profile alignment

**DOI:** 10.1515/jib-2019-0091

**Published:** 2020-06-29

**Authors:** Hafida Bouziane, Abdallah Chouarfia

**Affiliations:** Département d’Informatique, Université des Sciences et de la Technologie d’Oran Mohamed Boudiaf, USTO-MB BP 1505, El M’Naouer, 31000, Oran, Algeria

**Keywords:** gene ontology terms, gram-negative bacteria, gram-positive bacteria, multi-label learning, profile alignment, subcellular localization prediction

## Abstract

To date, many proteins generated by large-scale genome sequencing projects are still uncharacterized and subject to intensive investigations by both experimental and computational means. Knowledge of protein subcellular localization (SCL) is of key importance for protein function elucidation. However, it remains a challenging task, especially for multiple sites proteins known to shuttle between cell compartments to perform their proper biological functions and proteins which do not have significant homology to proteins of known subcellular locations. Due to their low-cost and reasonable accuracy, machine learning-based methods have gained much attention in this context with the availability of a plethora of biological databases and annotated proteins for analysis and benchmarking. Various predictive models have been proposed to tackle the SCL problem, using different protein sequence features pertaining to the subcellular localization, however, the overwhelming majority of them focuses on single localization and cover very limited cellular locations. The prediction was basically established on sorting signals, amino acids compositions, and homology. To improve the prediction quality, focus is actually on knowledge information extracted from annotation databases, such as protein–protein interactions and Gene Ontology (GO) functional domains annotation which has been recently a widely adopted and essential information for learning systems. To deal with such problem, in the present study, we considered SCL prediction task as a multi-label learning problem and tried to label both single site and multiple sites unannotated bacterial protein sequences by mining proteins homology relationships using both GO terms of protein homologs and PSI-BLAST profiles. The experiments using 5-fold cross-validation tests on the benchmark datasets showed a significant improvement on the results obtained by the proposed consensus multi-label prediction model which discriminates six compartments for Gram-negative and five compartments for Gram-positive bacterial proteins.

## 1 Introduction

Proteins are key players in cell survival and damage and their presence in specific cell sites reflects the nature of their biological function. Protein subcellular localization (SCL) knowledge is thus valuable for protein function elucidation which is crucial for drug design, discovery, and development. Once they are synthetized in the cytosol, proteins are directed to specific cell compartments called organelles to perform their proper biological functions. It is well known fact that two main phenomena are responsible for cell dysfunction or damage leading to serious diseases, protein misfolding and erroneous presence of proteins in cell compartments. Hence, to at least avoid protein subcellular mislocalization, accurate trafficking of proteins to their ultimate destinations is crucial [1]. Although the intense research efforts made to understand such mechanisms and their influence on the cell functional machinery, further investigations are still expected for full insight on the proteins behavior *in-vivo*. Such efforts depend on the development of new in silico methodologies as alternative to the costly and ardious wet-lab experiments which are sometimes impractical due to the nature of certain proteins. Today, with the rapid development of structural bioinformatics and sequential bioinformatics, computational methods have become essential in genomic and proteomic analyses. However, the major machine learning-based methods are facing two challenges, consisting in: (i) the presence of multi-location proteins which may be located in more than one organelle simultaneously and assigning proteins to a single location is a drastic simplification of reality [2], (ii) the imbalanced nature of learning datasets of annotated protein sequences as most learner systems exhibit bias towards the majority class while the minority class is generally of greatest interest. In order to make up the shortfalls and achieve desirable results, computational methods development for SCL prediction focuses on powerful individual learning models, ensemble models to take advantage of their combined strengths, and heterogeneous data integration. The pioneering methods predicted the SCL solely from the amino acid sequence such as the rule-based expert system PSORT-I developed by Nakai and Kanehisa [3], [4] and the probabilistic model proposed by Horton and Nakai [5]. Thereafter, different classification algorithms have been used to further improve the performance. Among these algorithms, k-Nearest Neighbor (k-NN) [6], [7], binary Decision Tree (DT) [8], Naive Bayesian (NB) classifier [9], [10], [11], Artificial Neural Networks (ANNs) [12], [13], [14], [15], Support Vector Machines (SVMs) [16], [17], [18], [19], [20], [21], [22], [23], Hidden Markov Models (HMMs) [24], and Bayesian networks [9], [25]. Since these works, many systems using a variety of machine learning techniques have been proposed achieving varying degrees of success. They were specialized for specific organisms and certain localization sites, but no significant improvements over the k-NN algorithm were reported until the burt of the new generation methods based on hybrid models and fusion approach [26–33] taking into account both protein sequence and structure charachteristics. They are categorized as sorting signals-based, composition-based and homology-based methods. The first category includes MitoProt [33], PSORT-II [6], ChloroP [34], TargetP [13], iPSORT [35], PSORT-B [25], and NucPred [36]. The second category includes Sub-Loc [16], Esub8 [24], ESLpred [37], pSLIP [38], AAIndexLoc [39], ngLoc [112], YLoc [11], and BaCelLo [41]. The third category includes phylogenetic profiling based methods [40, 42] and sequence homology-based methods such as GOASVM [23] and SCLpredT [43]. Recently, protein–protein interaction [44], [45], [46], gene expression levels [23], [26], [47], [48], [49] and textual information [40] has been also integrated to infer the subcellular locations exploiting the available databases of proteins with known localization [50]. SCL prediction based on deep learning methods has also emerged due to their ability to learn high-level features [30], [51], [52], [53], [54], [55]. The success achieved by using both composition and homology information [56], [57], [58], [59], [60], [61] has led to a plethora of methods using different strategies to improve the prediction quality. However, the best performing SCL systems reported to date are mainly based on multi-label learning and Gene Ontology (GO) annotation which has revolutionized the way to represent biological knowledge so as to be computationally accessible [48], [62], [63]. Basically, GO concept describes the roles of genes across different organisms and allows functional inference for newly discovered genes. The established collection of terms adopted and standardized by the GO Consortium1
https://geneontology.org/. [64] are derived from experimental and electronic annotations. They are organized in three distinct sub-ontologies that represent gene/protein functions aspects: Molecular Function (MF), Biological Process (BP), and Cellular Component (CC). Molecular function corresponds to activities that can be performed at the molecular level, such as catalytic, binding, or transporter activities. A biological process corresponds to pathways and programs involved in. A cellular component is either the cellular environment or extracellular region where the activity is executed. Each category of GO terms is organized as a directed acyclic graph (DAG) with terms as nodes and relationships as edges. There is a structured hierarchy with defined semantic relationships between terms, where each term can have relationships to several parent and child terms. High level terms are more general and low level terms are more specific than their respective parent terms. The most ubiquitous relationships are: “is-a” which describes the fact that child term is an instance of parent and “part-of” which shows that child term is a component of parent. Each GO term has a unique alphanumeric identifier where functional assignment source is indicated in the form of Evidence Code2
https://www.geneontology.org/doc/GO.Evidence.html. which might be experimental, computational or automatic-assignment evidence. In this paper we investigate the effect of GO annotation and profile alignment on SCL prediction of Gram-negative and Gram-positive bacterial proteins using multi-label learning. These microscopic unicellular prokaryotes distinguished by the lack of cell nucleus play a critical role in health problems. Despite their beneficial effect, they are mostly pathogenic and source of many diseases in humans. There are only a few methods that concentrate on this specie by tackling the SCL prediction problem using both multi-label learning and GO terms [32], [], so, here we tried to estimate how much improvement over the traditional pseudo amino acid composition (PseAAC) [72] might be provided by using position-specific scoring matrix (PSSM) profiles, GO terms and both. Generally, GO terms are retrieved by querying protein accession number against GOA 3
https://www.ebi.ac.uk/GOA/index. database for annotated proteins or using either InterProScan4
https://www.ebi.ac.uk/interpro/search/sequence/. [73] to scan query proteins for significant matches against the InterPro5
https://www.ebi.ac.uk/interpro. protein signature databases or BLAST [74] to obtain accession numbers of homologous proteins as the searching keys for uncharacterized proteins. Here, BLAST is used as baseline for similarity search to transfer the GO terms of homologous proteins to target proteins and to infer PSSM profiles. Our proposed model can predict five and six distinct locations on Gram-positive and Gram-negative bacterial proteins, respectively. In order to achieve such a goal, we tried to follow the well-established process of Chou’s 5-steps rule [75] for developing fast and reliable computational methods for genomic or proteomic analysis and drug development [76], [77], [78], [79], [80]. As it is explicitly described in review papers [81], [82], [83] and Wikipedia, our major efforts were thus devoted to: (i) collecting benchmark datasets from experimentally validated protein sequences to train and test the method; (ii) using an effective mapping of protein samples from sequential to vectorized representation; (iii) developing a powerful SCL prediction model for Gram-positive and Gram-negative bacterial proteins, exploiting diversity in both feature and decision spaces; (iv) performing cross-validation tests with appropriate measures to effectively evaluate the prediction model performance; (v) implementing a user-friendly web-server for the proposed prediction model to make it publicly available since such tools allow to avoid going through the complicated scientific and mathematical formulas, and represent the future direction for developing practically more useful predictors. Below, we describe how we performed these steps to obtain our final prediction model.

The rest of the paper is organized as follows. First we present the proposed framework. Next, we briefly describe the benchmark datasets and the evaluation methodologies adopted and then, we summarize the experiments and the results obtained. Finally, in the last section we conclude and present some future research plans.

## 2 Method

**Figure 1:**
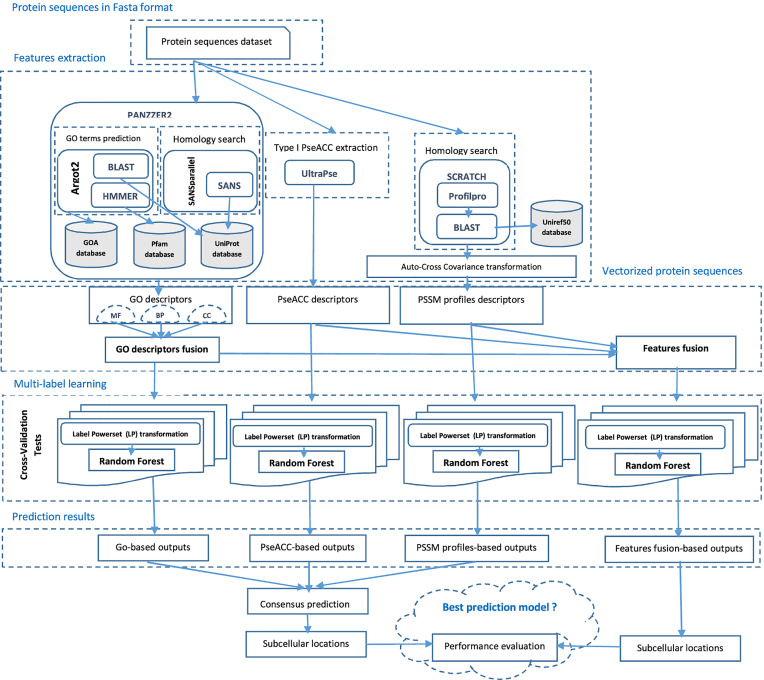
Flowchart for the proposed prediction model for Gram-positive and Gram-negative bacterial proteins subcellular localization. Firstly, protein sequences datasets were collected from the published database. Secondly, they were filtered out and preprocessed using different strategies to obtain a fixed size feature vector representation that can be fed into the learning model. Thirdly, the resulting encoded feature vectors were independently put into the multi-label learning model-based on Label Powerset (LP) transformation to produce independent prediction scores using Random Forest (RF) ensemble method as base classifier. Once optimum performance scores were calculated by using 5-fold cross-validation tests, the final prediction model is built.

Traditional supervised learning algorithms learn from examples associated with only one single label either for binary or multi-class classification. However, many real-world problems deal with data that does not fail in this category and are referred to as multi-label learning problems, due to the nature of their training examples which are associated with multiple labels simultaneously. To deal with such situation, two approaches are adopted namely, algorithm adaptation and problem transformation. The first approach modifies or extends the existing algorithms to obtain dedicated versions, taking into account the multi-label nature of the samples. Whereas, the second and the most used approach transforms the original data so as to be able to apply the traditional algorithms. Two strategies are applied for the latest, Binary Relevance (BR) and Label Powerset (LP) transformations. The first model consisting in class binarization principle applies the traditional one-vs-all approach to transform the original multi-label problem to several bi-class sub-problems to apply binary classifiers and combines the predictions. The second model transforms the multi-label problem into a multi-class problem by giving the set of labels associated to each instance, a class identifier to apply any multi-class classifier. BR is relatively a naive approach since each label is learned independently, assuming label independence. However, in multi-label learning from imbalanced data, which is inherent to SCL prediction, it is important to exploit label relations or dependency to improve the performance. Although, many studies focused on label co-occurrence and correlation to improve the prediction quality, no approach works consistently better on all kinds of multi-label datasets. In this study, we tried to capture the correlation information among labels by using Label Powerset (LP) strategy and the ensemble method Random Forest [84] as baseline classifier. The flowchart in Figure 1 describes the main framework of our proposed method for predicting Gram-positive and Gram-negative bacterial proteins SCL.

### 2.1 Fusion strategy

In traditional supervised learning, a sample is represented by an instance or feature vector and its associated single class label. Let us denote by $X\subseteq R^{d}$ a d-dimensional feature space and y a finite set of *Q* class labels $\left\{y_{1},\;y_{2},\;\cdots ,\;y_{Q}\right\}$. The goal is to learn a function f:x→y from a set of instances $D=\left\{\left(x_{1},\;y_{1}\right),\;\left(x_{2},\;y_{2}\right),\;\ldots ,\;\left(x_{N},\;y_{N}\right)\right\}$, where $x_{i}\in x$ is an instance and $y_{i}\in y$ is the known label of $x_{i}$. In multi-label learning, the number of all label vectors is generally 2^Q^. Each label $y_{i}\in y\subseteq {\left\{1,\;0\right\}}^{Q}$, where $y_{i}\left[j\right]=1$ if and only if the instance $x_{i}$ is associated with the *j*th label, 1≤j≤Q. Given the multi-label training set D, the task of multi-label learning consists to learn a predictor *h* from D, $h:x\to 2^{y}$ mapping from the instance space to the label space. For each instance (x,y) in test, $\tilde{y}=h\left(x\right)$ is the predicted label and $c\left(y,\;\tilde{y}\right)$ the cost or penalty of predicting *y* as $\tilde{y}$, such value might be quantified using the evaluation metrics given in Section 3.2. In Label Powerset the transformation process only modifies the label space, considering the set of distinct unique subsets of labels present in the original training set [85]. In the novel label space the multi-class label $Y=y_{i,\;j,\;\ldots ,\;Q}$ means that the respective instance is labeled with the conjunction $y_{i}\wedge y_{j}\wedge \ldots \wedge y_{Q}$. The first prediction model is based on PSSM profiles and the second on GO terms. As the two best individual prediction models will probably provide a reduced set of multi-label outputs, our goal is to improve multiple locations assignation to query proteins. The outputs of the consensus prediction model consist on the union of the individual models predictions. Suppose ${\tilde{Y}}^{\text{PSSM}}={\tilde{y}}_{i,\;j,\;\ldots ,\;Q}^{\text{PSSM}}$ be the predicted multi-class label obtained using PSSM profiles and ${\tilde{Y}}^{\text{GO}}={\tilde{y}}_{i,\;j,\;\ldots ,\;Q}^{\text{GO}}$ using the predicted GO terms, respectively. The multi-class label obtained by the consensus prediction is as follows:

(1) $${\tilde{Y}}^{\text{Consensus}}={\left({\tilde{y}}^{\text{PSSM}}\vee {\tilde{y}}^{\text{GO}}\right)}_{i,\;j,\;\ldots ,\;Q}$$

The consensus prediction for each query protein is obtained by the conjunction (bitwise OR) of its respective predicted single class labels. For example, in the case of Gram-negative bacterial proteins, where the number of predicted subcellular locations is 6 (see Table 1), the prediction model decision is obtained as follows:

$$\begin{array}{c} {\tilde{Y}}^{\text{PSSM}}=\left[0,\;0,\;0,\;0,\;1,\;0\right]\;which\;represents\;S\;\left(Extracellular\right)\;location\\ {\tilde{Y}}^{\text{GO}}=\left[1,\;0,\;0,\;0,\;0,\;0\right]\;which\;represents\;I\;\left(Inner\;Membrane\right)\\ {\tilde{Y}}^{\text{Consensus}}=\left[1,\;0,\;0,\;0,\;1,\;0\right]\;which\;is\;represented\;by\;S/I\;\left(Extracellular\;and\;Inner\;Membrane\right) \end{array}$$

**Table 1:** Prokaryotic benchmark datasets statistics. Code column indicates the subcellular location representation in our predictive model. Gram-negative bacteria have five major subcellular localization sites, namely, the cytoplasm, the periplasm, the inner membrane, the outer membrane, and the extracellular space, whereas Gram-positive bacteria do not have an outer cell membrane. However in these benchmark datasets cell wall is absent in Gram-negative dataset and in Gram-positive bacteria, we observe the lack of periplasm proteins.

No	Subcellular location	Code	Proteins count	
			Gram negative	Gram positive
1	Cytoplasm	C	4,152	349
2	Extracellular	S	272	290
3	Inner membrane	I	1,415	1,779
4	Outer membrane	O	346	–
5	Periplasm	P	422	–
6	Cell wall	W	–	34
7	Vacuole	V	10	4
Multiple localizations			39	8
Total			6,578	2,448

The query protein is thus predicted in both extracellular region and inner membrane.

### 2.2 Protein sequence representation

There is no doubt that protein sequence representation influences protein subcellular location prediction performance. SCL prediction methods have tried different protein sequence and structure properties to improve the prediction quality but it appears that incorporating GO information is decisive for SCL prediction quality. In the following subsections, we describe protein sequences representation step which has led to several versions of the benchmark datasets.

#### 2.2.1 Pseudo amino acid composition

In computational biology, an important but challenging step is how to represent a biological sequence by a discrete model or a vector that captures its key features without losing sequence-order information, since most of the existing machine-learning algorithms can only handle fixed-length numerical vectors [86], [87]. When dealing with protein sequences, the simplest way to characterize a protein by a fixed-length numerical vector is to extract the information of the protein sequence from the entire amino acid sequence, especially when the protein does not have significant homology to annotated proteins. K.C. Chou proposed PseAAC (pseudo amino acid composition) which has been extensively used in protein-related prediction systems as it has been introduced to enhance the power of the conventional discrete amino acid composition (AAC) which consists of 20 components representing the occurrence frequencies of the 20 naturally occurring amino acids in the sequence. PseAAC incorporates both the sequence order and the length effect [72]. Due to the great success of such representation model for protein sequences in different areas of computational biology, the concept of PseAAC has been extended to represent nucleotide sequences with the concept PseDNC (pseudo-dinucleotide compositions) and the concept of PseKNC (Pseudo K-tuple Nucleotide Composition) [88]. Many Web-servers and stand-alone programs are now available to generate such features and any other desired features for protein/peptide sequences such as PseAAC-Builder6
https://pseb.sourceforge.net/. [89], propy7
https://code.google.com/p/protpy/downloads/list. [90], PseAAC-General8
https://pseb.sourceforge.net/. [91], Pse-in-One9
https://bioinformatics.hitsz.edu.cn/Pse-in-One/. [92] and its very powerful updated version Pse-in-One 2.010
https://bioinformatics.hitsz.edu.cn/Pse-in-One2.0/. [93], and UltraPse11
https://github.com/pufengdu/UltraPse. software [94] which allows all possible sequence representation modes for user-defined sequence types. Here, UltraPse source code has been downloaded and run locally on our ubuntu platform to extract pseudo amino acid composition (Type I General PseAAC) for each protein sequence in the benchmark datasets.

#### 2.2.2 Generation of PSSM profiles

Protein sequences have been represented by position-specific scoring matrix (PSSM) profiles which reflect the frequencies of each amino acid residue in a specific position of a multiple alignment. To obtain such mapping, we used PROFILpro release 1.1 integrated in SCRATCH12
https://scratch.proteomics.ics.uci.edu/. server [95] which performs with BLAST version 2.2.26 [74] and a non-redundant UniRef5013
ftp.uniprot.org/pub/databases/uniprot/uniref/uniref50. database (clustered sets of protein sequences that show 50% sequence identity) as the search database. Protein PSSM features were computed by setting the number of iterations to three (−*j*3) and the inclusion e-value to 0.001 (−h0.001). Each feature vector is a 20 × *L* dimension, where *L* is the length of the protein sequence. To map the obtained feature vectors of varying lengths to fixed length vectors while preserving the local sequence-order information, auto-cross covariance tranformation (ACC) has been applied [96] which is provided by Pse-in-One 2.0 and protr14
https://cran.r-project.org/web/packages/protr/index.html. R package [97]. Each protein sequence is thus represented by a numeric vector of length $lg\text{*}20^{2}$, where *lg* is the distance between one amino acid residue and its neighbor along the protein sequence. To describe the ACC transformation, let us denote by *p*_*i,j*_ the probability (score) of amino acid i occurring at the position j in the PSSM, if we consider each amino acid as one property and the PSSM as the time sequences of all properties. ACC transformation converts the PSSM of different lengths into a fixed-length vector by measuring the correlation between each pair of properties. It first builds two signal sequences, and then calculates the correlation between them. Let us denote by ${\bar{p}}_{i}$ the average score for amino acid *i* along the whole sequence, expressed by:

(2) $${\bar{p}}_{i}=\frac{1}{L}\sum_{j=1}^{L}p_{i,\;j}$$

ACC results in two kinds of variables: auto covariance (AC) between the same property, and cross covariance (CC) between two different properties. The AC variable measures the correlation of the same property between two residues separated by a distance of lag along the sequence and can be calculated as follows:

(3) $$AC\left(i,\;lag\right)=\frac{1}{L-lag}\sum_{j=1}^{L-lag}\left(p_{i,\;j}-{\bar{p}}_{i}\right)\left(p_{i,\;j+lag}-{\bar{p}}_{i}\right)$$

where *i* is one residue of the protein sequence of length *L*.

In this way, the number of AC variables is 20 × *lg*, where 20 corresponds to the number of columns of the PSSM and lg is the maximum value of lag (lag=1,2,⋯,lg).

The CC variable measures the correlation of two different properties between two residues separated by *lag* along the sequence as follows:

(4) $$CC\left(i,\;j,\;lag\right)=\frac{1}{L-lag}\sum_{k=1}^{L-lag}\left(p_{i,\;k}-{\bar{p}}_{i}\right)\left(p_{j,\;k+lg}-{\bar{p}}_{j}\right)$$

where *i* and *j* are two different amino acids and ${\bar{p}}_{i}$ (${\bar{p}}_{j}$) is the average score for amino acid *i* (*j*) along the sequence.

Each protein sequence is thus represented as a vector of ACC-derived variables as combination of AC and CC variables. Here, the parameter value *lg* is set to one to reduce the computational time for a larger dataset, which is considered as a default parameter of our predictive model. However, it is worth noticing that taking into account the amino acids neighboring effect may be able to improve the prediction quality, so further investigations are required to evaluate the contribution of *lg* value to the performance of the proposed prediction model.

#### 2.2.3 Gene ontology terms prediction

GO terms prediction is also an active area of research for in silico functional annotation. Uncharacterized proteins do not have accession numbers since they are not repertorized in databases yet and hence direct GO terms retrieval from GOA database is not possible. Such is the case, of newly-discovered, synthetic and hypothetical proteins [98]. Computational methods try to annotate these proteins by transferring GO terms from annotated proteins by sequence or structural similarity [99]. Furthermore, it is assumed that proteins sharing common primary and secondary structures are generally located in the same cellular regions [100], [101], [102], [103]. Here, the prediction system takes as input a list of sequences in FASTA format and predicts GO terms using PANZZER215
https://ekhidna2.biocenter.helsinki.fi/sanspanz/. (Protein ANNotation with Z-score) [104], [105] which uses SANSparallel16
https://ekhidna2.biocenter.helsinki.fi/cgi-bin/sans/sans.cgi. [106] based on SANS (suffix array neighborhood search) algorithm [107] to perform high-performance and fast homology searches in the UniProt17
https://www.uniprot.org/. database instead of BLAST. The benchmark datasets have been treated in batch mode to obtain both GO annotations and free text protein descriptions (DE) files. Generally, multiple GO terms can be assigned to each query protein which can induce a strong statistical redundancy, while only a relatively small number of unique GO terms is essential for protein annotation [108]. To prevent potential redundancy and select correct annotations PANZZER2 includes implementations of the scoring functions from PANZZER (its basic version), Blast2GO18
https://www.blast2go.com/
 [109], [110] and Argot219
https://www.medcomp.medicina.unipd.it/Argot2-5/. (Annotation Retrieval of GO Terms) [63]. The latest exploits a combined approach based on the clustering process of GO terms dependent on their semantic similarities and a weighting scheme which assesses retrieved hits sharing a certain degree of biological features with the sequence to annotate. Where, hits may be obtained by BLAST with UniProt as reference database or HMMER20
https://hmmer.janelia.org. with Pfam21
https://pfam.xfam.org/. using a recent release of UniProtKB-GOA database [111]. Both Blast2GO and Argot are suitable for non-model species, however, our analysis in this study was based on Argot predictions since it has been revisited to increase both accuracy and precision by using an improved weighting scheme, Pfam models and new releases of reference databases. For each query protein sequence a list of scored and ranked GO terms is provided. The GO term set consists of three subsets: molecular function (MF), biological function (BP), and cellular component (CC). Once the relevant GO subspace is obtained, each subset is processed in order to obtain numerical feature vectors of probability estimates using PPV (Positive Predictive Value) which is the normalized prediction score between 0 and 1. We adopted such strategy for the sake of comparison with the common practice that consists to construct feature vectors by using 0/1 value to represent the presence and absence of the predefined GO terms or the frequency of occurrences of GO terms [23].

## 3 Experimental design

In this section, we present the experimental design used to evaluate our SCL prediction model. We first describe the benchmark datasets collected from the literature. The coverage of our proposed model is directly related to the available annotation terms in the datasets used for training, taken as class labels. Each class label is a binary encoded vector of length equal to the number of distinct locations. For protein sequences annotated by two or more locations, multiple locations are encoded by summing up (bitwise OR) each corresponding binary vector. Some evaluation measures typically applied in multi-label learning based prediction are summarized in this section.

### 3.1 Benchmark datasets

**Figure 2:**
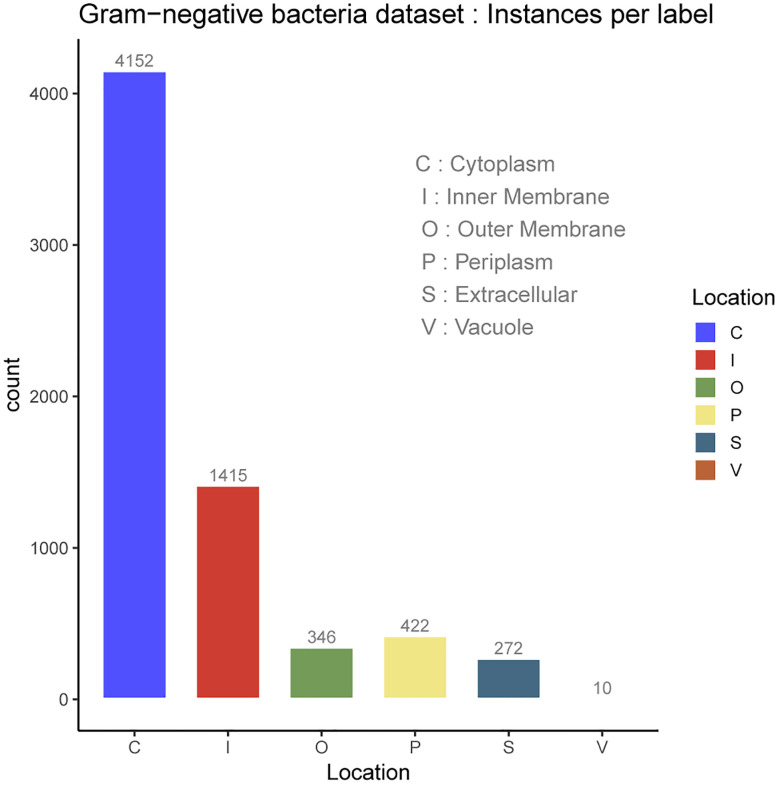
Gram-negative bacteria dataset.

**Figure 3:**
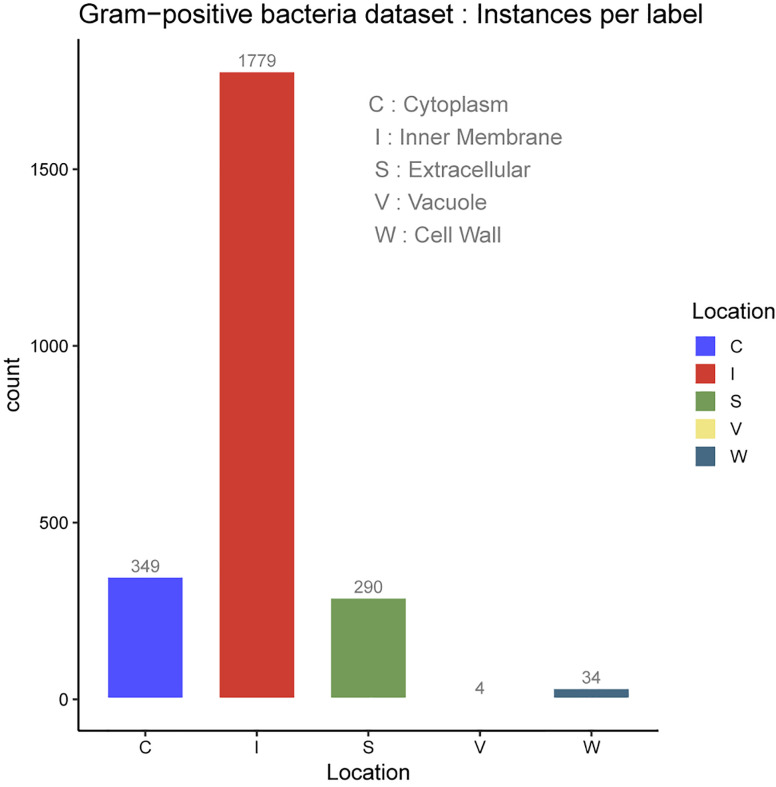
Gram-positive bacteria dataset.

In the protein SCL dataset, a protein might be associated with a set of SCL labels related to the cell type. Prokaryotic cell is typically composed of a cell wall which protects the cell and gives shape, a cell membrane which separates the intracellular environment from the extracellular space which is outside the plasma membrane, and the most abundant cytoplasm where the major cellular processes are performed. Some prokaryotic cells produce gas vacuoles named gas vesicles. One distinguishes Archaea and Bacteria cells, the latest are divided in two broad categories according to their cell wall, Gram-positive, and Gram-negative. Gram-negative bacteria have five major SCL sites, which are the cytoplasm, the periplasm, the inner membrane and the outer membrane. The inner membrane separates the cytoplasm from the periplasm. The outer membrane protects the cell against some antibiotics, and the extracellular space. The outer membrane is absent in Gram-positive bacteria which allows antibiotics reception. The volume of periplasm is much smaller than in Gram-negative bacteria and it is characterized by a thicker cell wall. Our SCL prediction model has been benchmarked on two independent datasets of experimentally determined annotations on SCL. They were collected from curated set of bacterial protein sequences Gram-positive and Gram-negative, taken from the Swiss-Prot database release of May 17th 2011 [112], available here. These datasets contain protein sequences having less than 98% sequence identity to reduce sequence redundancy. Here, protein datasets have been filtered out so as to only consider protein sequences with the 20 standard amino acids and excluded sequences containing *X* symbol because of their ambiguity. The number of proteins in each main localization obtained after the filtering process are summarized in Table 1 for Gram negative and Gram positive bacteria datasets. The class referred to as Vacuole contains the gas Vesicle proteins incorporated in both datasets, such class or proteins appears only in certain prokaryotic organisms. Gram-positive and Gram-negative bacteria are chiefly differentiated by their cell wall structure, Table 1 lists the number of proteins in different localization sites in the datasets and Figure 2 and Figure 3 report the datasets statistics. The two benchmark datasets are imbalanced since the distributions of the proteins in different locations is uneven. The majority of Gram-negative bacterial proteins are located in the cytoplasm, the inner membrane and the periplasm, whereas the Gram-positive bacterial proteins are located in the cell inner membrane, the cytoplasm and the extracellular space. As we can see, the number of protein samples in Vacuole (V) which represent the gas Vesicle location is 10 in Gram-negative dataset and only 4 in Gram-positive dataset, which represents the minority class. It is worth noticing that such class is rare in bacterial proteins, it is present in aquatic and marine bacteria, while it represents an important class in archaea and planktonic species. The same trend is observed for the cell wall (W) compartment. Cytoplasm is the most abundant class in Gram-negative dataset whereas it is significantly dominated by Inner membrane (I) class in Gram-positive dataset. In these benchmark datasets cell wall is absent in Gram-negative dataset and in Gram-positive bacteria, we observe the lack of periplasm proteins which indicates that our predictive model will not predict the cell wall location for Gram-negative bacteria and periplasm location for Gram-positive bacteria.

**Figure 4:**
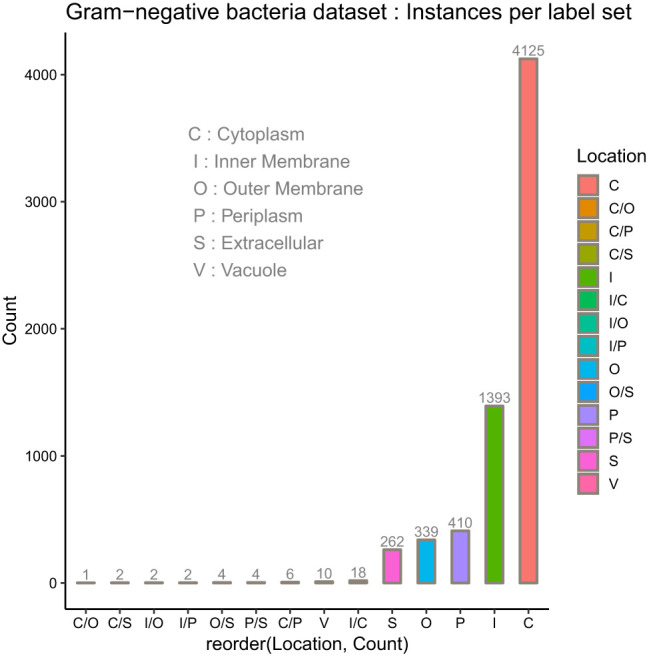
Observed localization sites of proteins in Gram-negative bacteria dataset.

**Figure 5:**
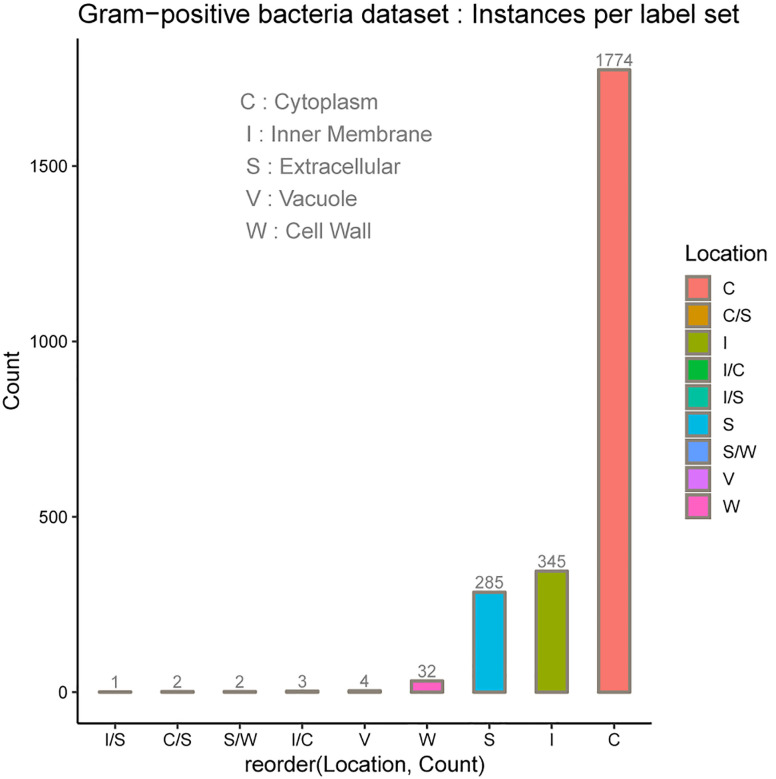
Observed localization sites of proteins in Gram-positive bacteria dataset.

Multiple sites proteins are shown in Figure 4 for Gram-negative bacteria and Figure 5 for Gram-positive bacteria. As it can be observed, multiple locations proteins are limited to a pair sites which is generally the case of the majority of multiple sites proteins [2].

### 3.2 Performance measures

The performance evaluation of multi-label learning based prediction models needs specific metrics, since each instance could be associated with two or more labels simultaneously. Various meaningful metrics have been used in the literature such as example-based metrics and label-based metrics [113], [114]. The first category evaluates the generalization performance on each test instance and returns the average value for the entire test set, whereas the second category proceeds first on each class label separately, and then the average value is calculated across all class labels. The latest measures are derived from the four common values used in binary classification, namely TP (true positive), FP (false positive), TN (true negative), and FN (false negative). The term macro is used for a measure such as recall, precision, F1 score derived by assuming equal importance for each label while micro corresponds to that derived by assuming equal importance for each example [115]. In our study, we adopted both example-based and label-based metrics. They are implemented in both mldr22
https://cran.r-project.org/web/packages/mldr/index.html. [116] and utiml23
https://cran.r-project.org/web/packages/utiml/index.html. [117] R packages. Given a test instance $x_{i},\;i=1,\;\ldots ,\;N$, y the set of all labels, $Y_{i}\subseteq y$ the set of true labels and ${\tilde{Y}}_{i}$ the set of predicted labels for $x_{i}$, the metrics are thus described in the following subsections.

#### 3.2.1 Example-based measures

*Accuracy score* computes the percentage of correctly predicted class labels among all predicted and true class labels for each instance, it is defined as follows:

(5) $$Accuracy\;score=\frac{1}{N}\sum_{i=1}^{N}\frac{{\left\|Y_{i}\cap^{\text{​}}{\tilde{Y}}_{i}\right\|}_{1}}{{\left\|Y_{i}\cup^{\text{​}}\tilde{Y}\right\|}_{1}}$$

It is important to note that using Accuracy metric alone may mislead the analysis when dealing with imbalanced data and high scores do not necessarily indicate good performance.

**Precision** is the proportion of TP examples from all the examples predicted as positive.

(6) $$Precision=\frac{1}{N}\sum_{i=1}^{N}\frac{{\left|\left|Y_{i}\cap^{\text{​}}{\tilde{Y}}_{i}\right|\right|}_{1}}{{\left|\left|{\tilde{Y}}_{i}\right|\right|}_{1}}$$

**Recall** is the proportion of TP examples predicted as positive.

(7) $$Recall=\frac{1}{N}\overset{N}{\underset{i=1}{\sum }}\frac{{\left|\left|Y_{i}\cap^{\text{​}}{\tilde{Y}}_{i}\right|\right|}_{1}}{{\left\|Y_{i}\right\|}_{1}}$$

**F1 score** is the harmonic mean between Precision and Recall, expressed as follows:

(8) $$F1\;score=\frac{1}{N}\sum_{i=1}^{N}\frac{{\left\|2Y_{i}\cap^{\text{​}}{\tilde{Y}}_{i}\right\|}_{1}}{{\left\|Y_{i}\right\|}_{1}+{\left\|{\tilde{Y}}_{i}\right\|}_{1}}$$

**Subset-accuracy**

(9) $$Subset\_accuracy=\frac{1}{N}\sum_{i=1}^{N}I\left(Y_{i}={\tilde{Y}}_{i}\right)$$

where I function is defined as I(true) = 1 and I(false) = 0. This metric takes into account only exact matches and by ignoring partially correct matches, it will not be able to recognize nearly exact prediction from totally incorrect prediction. While, it would be interesting to know if an example is correctly assigned to at least one of the labels it belongs to, especially when dealing with imbalanced data.

**Hamming-Loss** gives the fraction of labels that are incorrectly predicted. It is the widespread evaluation metric in multi-label learning systems, expressed as:

(10) $$Hamming-loss=\frac{1}{N}\sum_{i=1}^{N}\frac{1}{Q}\sum_{j=1}^{Q}{\left\|Y_{i}\left[j\right]\neq {\tilde{Y}}_{i}\left[j\right]\right\|}_{1}$$

**Rank-loss** evaluates the average proportion of label pairs that are incorrectly ordered for an example. It is defined as follows :

(11) $$Rank-loss=\frac{1}{N}\sum_{i=1}^{N}\sum_{Y_{i}\left[j\right]>Y_{i}\left[l\right]}\left(\left[\left[{\tilde{Y}}_{i}\left[j\right]<{\tilde{Y}}_{i}\left[l\right]\right]\right]+\frac{1}{2}\left[\left[Y_{i}\left[j\right]={\tilde{Y}}_{i}\left[l\right]\right]\right]\right)$$

The higher the value of Accuracy and F1 score, the better the performance of the learning algorithm and the smaller the value of Hamming loss and Rank loss, the better the performance. The Area Under the ROC Curve (AUC) is also used since it is a good indicator of performance, especially when dealing with multi-label learning problems.

#### 3.2.2 Label-based measures

As it has been mentioned above, these metrics are obtained for all labels by either maco-averaging or micro-averaging.

**Macro_precision** is defined by the fraction of the number of TPs by the number of both TPs and false positives for the label *y*_*j*_ considered as a binary class.

(12) $$Macro\_precision=\frac{1}{Q}\sum_{j=1}^{Q}\frac{TP_{j}}{TP_{j}+FP_{j}}$$

**Recall** is defined by the fraction of the number of TPs by the number of both TPs and false negatives for the label *y*_*j*_.

(13) $$Macro\_recall=\frac{1}{Q}\sum_{j=1}^{Q}\frac{TP_{j}}{TP_{j}+FN_{j}}$$

**Macro_F1** score is the harmonic mean between Macro_precision and Macro_recall, expressed as follows:

(14) $$Macro\_F1\;\;score=\frac{2\text{*}Macro\_precision\text{*}Macro\_recall}{Macro\_precision+Macro\_recall}$$

**Micro_precision**

(15) $$Micro\_precision=\frac{\sum_{j=1}^{Q}TP_{j}}{\sum_{j=1}^{Q}\left(TP_{j}+FP_{j}\right)}$$

**Micro_recall**

(16) $$Micro\_recall=\frac{\sum_{j=1}^{Q}TP_{j}}{\sum_{j=1}^{Q}\left(TP_{j}+FN_{j}\right)}$$

**Micro_F1 score** is the harmonic mean between Micro_precision and Micro_recall, expressed as follows:

(17) $$Micro\_F1\;\;score=\frac{2\text{*}Micro\_precision\text{*}Micro\_recall}{Micro\_precision+Micro\_recall}$$

### 3.3 Results and discussion

This section presents the results of the study and a synthesis of the experiments carried out, starting by the homology-based GO extraction. The studied SCL prediction models have been assessed using cross-validation tests to infer the best ensemble model with a special interest to multiple sites proteins as it is illustrated in Figure 1.

#### 3.3.1 Extracted GO terms

First we extracted from the learning datasets three sets of distinct GO terms which are the top ranked GO terms provided by PANNZER2, corresponding to the three sub-ontology molecular function (MF), biological process (BP), and cellular component (CC) by removing the repetitive GO terms. Then the feature vector has been constructed for each protein given in FASTA format from the union of these essential GO terms for two reasons: (i) to increase the possibility of getting at least one GO term for protein encoding to reduce the scenario where annotation is totally absent, (ii) to enhance the prediction quality since it has been found that not only CC GO terms are indicative of cellular component but also both MF and BP GO terms contribute to the final predictions. Many studies characterized a protein by a feature vector of 0/1 values indicating whether the protein is annotated with a predefined GO term or not, or the frequency of such GO term in [118]. Here, we investigated both 0/1 representation and the GO term representation by the PPV (positive predictive value) score assigned by Argot2 predictor. The numbers of GO terms in the CC, MF, and BP sub-ontologies for each benchmark dataset are reported in Table 2.

**Table 2:** Extracted GO terms statistics for the three components of GO namespace, namely, Molecular Function (MF), Biological Process (BP), and Celullar Component (CC).

Dataset	GO terms count		
	MF	BP	CC
Gram negative	1,140	1,316	223
Gram positive	570	667	116

In these experiments, a total of 2679 GO terms were selected for the Gram-negative bacterial dataset and 1353 GO terms for Gram-positive bacterial dataset. The number of BP GO terms is significantly larger than that from the other two sub-ontologies; however, our aim in this study is not to assess which of these specific GO terms are influential in the prediction but to combine them to ensure that each query protein has at least one GO term. To predict the subcellular locations both 0/1- and PPV values-based representations of GO terms are assessed to build the final best predictive model.

#### 3.3.2 Cross-validation tests

We adopted the cross-validation method to evaluate the generalization ability of our proposed prediction model against the other studied models since it is still a good validation method for large datasets. To do so, we performed 5-fold cross-validation by randomly dividing protein sequences of each benchmark dataset into five mutually exclusive parts of approximately equal sizes so as the model learns from four parts, and tests are made on the remaining part. Then, the process is repeated and evaluated for all five possible combinations. Firstly, we have evaluated all the models built using different features individually to investigate the impact of the features on the prediction quality, namely, PseAAC, PSSM profiles, and GO terms descriptors. Then, we evaluated the performance using these features fusion for SCL prediction. We therefore set out to test how well Random Forest (RF) and Support Vector Machine (SVM) [119], [120] would perform as baseline classifiers for the multi-label approach used, as SVM is often claimed to be the best at dealing with complex classification problems. The results reported in Supplementary material S1 show that SVM outperformed RF only in the case when it learned from PseACC protein features, while RF was the best model when using PSSM profiles and GO terms as feature vectors. Once, the baseline classifier was selected, we exploited each SCL prediction model to infer a consensus prediction so as to increase the possibility of obtaining additional multi-label outputs, since individual predictions provide a poor set of multi-label outputs. The results reported in Table 3 and Table 4 show the performance of each prediction model using 5-fold cross-validation tests on Gram-negative and Gram-positive bacteria respectively. A total of eight prediction models has been obtained using PseAAC features, PSSM profiles, GO terms with both 0/1-, and PPV-based representations, fusion of all the extracted features that we represented by *PseAAC* + *PSSM* + *GO*, fusion of only PSSM and GO features represented by *PSSM*+*GO*, and the fusion of the individual predictions by our proposed consensus approach, where Consensus _*PSSM*+*GO*_ stands for the consensus decision obtained from the individual predictions of PSSM profiles- and GO terms-based models and Consensus_*PseAAC*+*PSSM*+*GO*_, from all the three individual prediction models.

**Table 3:** Performance evaluation results of cross-validation tests on Gram-negative bacteria dataset for different predictions: pseudo-amino acid composition (PseAAC), position-specific scoring matrix (PSSM) profiles, gene ontology (GO) terms 0/1-based representation, GO terms PPV-based representation, features fusion, a consensus prediction using both GO terms and PSSM profiles outputs, and a consensus of PseAAC, GO terms and PSSM profiles outputs. Italic values correspond to the best predictive model.

Sequence features	Example-based metrics							Label-based metrics					
	Accuracy	Precision	Recall	F1 score	Subset_accuracy	Hamming-loss	Rank-loss	Macro_precision	Macro_recall	Macro_F1 score	Micro_precision	Micro_recall	Micro_F1 score
PseACC	0.882	0.884	0.882	0.883	0.880	0.039	0.117	0.873	0.656	0.732	0.884	0.879	0.881
PSSM profiles	0.940	0.941	0.940	0.940	0.937	0.020	0.060	0.919	0.815	0.860	0.941	0.936	0.939
GO terms 0/1	0.963	0.965	0.963	0.963	0.960	0.012	0.037	0.952	0.865	0.903	0.965	0.960	0.962
GO terms ppv	0.965	0.967	0.965	0.965	0.962	0.011	0.035	0.958	0.869	0.908	0.967	0.962	0.964
PseAAC+ PSSM+GO	0.951	0.953	0.951	0.952	0.949	0.016	0.048	0.938	0.835	0.880	0.953	0.948	0.951
PSSM+GO	0.951	0.954	0.951	0.952	0.949	0.016	0.048	0.939	0.850	0.890	0.954	0.948	0.951
Consensus_PseAAC +PSSM+GO_	0.920	0.920	0.986	0.941	0.858	0.030	0.043	0.852	0.930	0.884	0.855	0.984	0.915
Consensus_PSSM+GO_	*0.953*	*0.954*	*0.982*	*0.963*	*0.922*	*0.016*	*0.029*	*0.907*	*0.922*	*0.911*	*0.923*	*0.980*	*0.951*

**Table 4:** Performance evaluation results of cross-validation tests on Gram-positive bacteria dataset for different predictions: pseudo-amino acid composition (PseAAC), position-specific scoring matrix (PSSM) profiles, gene ontology (GO) terms 0/1-based representation, GO terms PPV-based representation, features fusion, a consensus prediction using both GO terms and PSSM profiles outputs, and a consensus of PseAAC, GO terms and PSSM profiles outputs. Italic values correspond to the best predictive model.

Sequence features	Example-based metrics							Label-based metrics					
	Accuracy	Precision	Recall	F1 score	Subset_accuracy	Hamming-loss	Rank-loss	Macro_precision	Macro_recall	Macro_F1 score	Micro_precision	Micro_recall	Micro_F1 score
PseACC	0.896	0.897	0.895	0.896	0.894	0.041	0.104	0.708	0.510	0.559	0.897	0.894	0.895
PSSM profiles	0.937	0.938	0.937	0.937	0.935	0.025	0.062	0.909	0.763	0.812	0.938	0.935	0.937
GO terms 0/1	0.959	0.959	0.958	0.959	0.957	0.016	0.041	0.952	0.785	0.791	0.959	0.957	0.958
GO terms ppv	0.962	0.962	0.961	0.962	0.960	0.015	0.038	0.927	0.803	0.819	0.962	0.960	0.961
PseAAC+PSSM+GO	0.947	0.949	0.947	0.948	0.946	0.020	0.052	0.931	0.772	0.824	0.949	0.946	0.947
PSSM+GO	0.948	0.950	0.948	0.949	0.946	0.020	0.051	0.920	0.822	0.851	0.950	0.947	0.948
Consensus_PseAAC+PSSM+GO_	0.924	0.924	0.978	0.941	0.871	0.033	0.050	0.855	0.858	0.839	0.871	0.977	0.921
Consensus_PSSM+GO_	*0.950*	*0.950*	*0.974*	*0.958*	*0.924*	*0.021*	*0.038*	*0.886*	*0.854*	*0.851*	*0.924*	*0.973*	*0.948*

**Figure 6:**
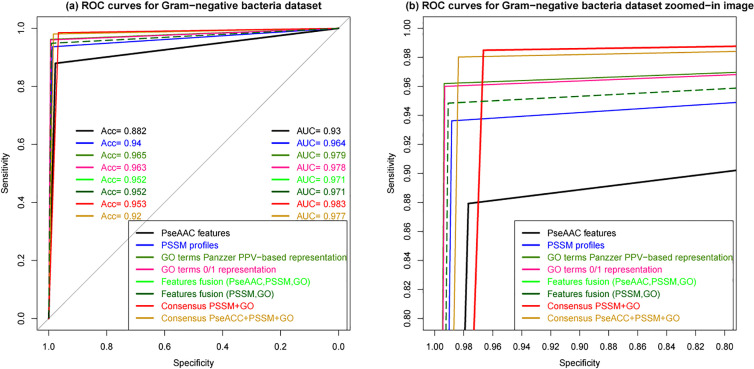
ROC curves of cross-validation tests on Gram-negative bacteria dataset for different predictions pseudo-amino acid composition (PseAAC), PSSM profiles, GO terms 0/1-based representation, GO terms PPV-based representation, features fusion, a consensus prediction using both GO terms and PSSM profiles outputs and a consensus of PseAAC, GO terms, PSSM profiles outputs. In (b) the zoomed-in image of the different curves.

**Figure 7:**
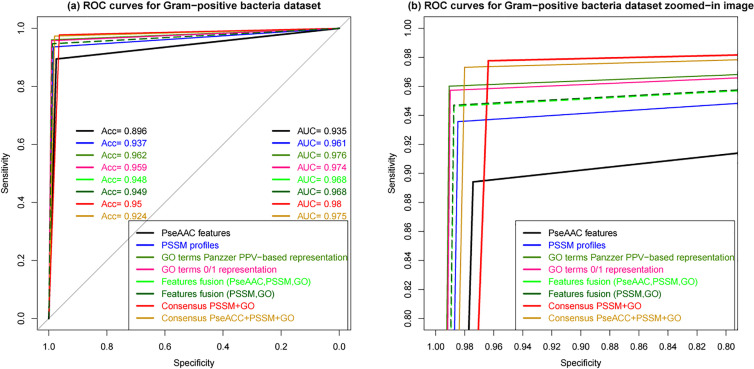
ROC curves of cross-validation tests on Gram-positive bacteria dataset for different predictions pseudo-amino acid composition (PseAAC), PSSM profiles, GO terms 0/1-based representation, GO terms PPV-based representation, features fusion, a consensus prediction using both GO terms and PSSM profiles outputs and a consensus of PseAAC, GO terms,PSSM profiles outputs. In (b) the zoomed-in image of the different curves.

From both Table 3 and Table 4, we observe that PSSM profiles-based prediction is significantly better than PseAAC-based prediction, whereas GO terms-based model clearly outperformed the two former individual prediction models. As it will be also observed, the two GO terms-based prediction models performed comparably well. However, the PPV-based representation of GO terms gave better performances than 0/1-based representation which suggests that substituting GO terms by 0/1 values do not truly reflect GO information. Moreover, features fusion by incorporating PseAAC, PSSM profiles and GO terms gave similar performances as by integrating only PSSM profiles and GO terms, which means that no significant gain in precision has been obtained in the presence of PseAAC features. It is clear that the most prominent improvement is achieved by the consensus model that combines the predictions of both PSSM profiles and GO terms-based individual models. The ROC curve provides a more realistic view of the prediction model performance, showing the specificity and sensitivity. The larger the area under the ROC curve, the better is the prediction quality. Here, the ROC curves in Figure 6 and Figure 7 depict well the performance of the individual prediction models built using different features and different ensemble models on Gram-negative bacteria and Gram-positive bacteria datasets. The difference between the studied prediction models is highly visible. It is clear that PseAAC-based model mostly performed rather weak, while the ROC curve of the Consensus prediction model that combines the decisions of both individual models based on PSSM profiles and GO terms is well above. Features fusion curves coincide, reflecting the poor influence of PseAAC features on the prediction quality, however such information would be useful when dealing with proteins that do not share any homology with annotated proteins.

For both datasets, the performances achieved by all eight prediction models are roughly the same. Capturing the most relevant biological features for protein characterization is crucial for prediction effectiveness. Here, it is clear that PseAAC features alone give inconsistent prediction, whereas, PSSM profiles and GO terms give significantly better predictive performances, especially the latest which outperformed the two others. Such result demonstrates that evolutionary information has a significant effect on the prediction performance and that GO information is a very good indicator for protein subcellular location. The results also show that in features fusion, when incorporating PseAAC features, no significant gain in performance is obtained since the ROC curves coincide. In addition, the prediction accuracy of the consensus model that considers PseAAC prediction model decisions declines for both Gram-positive and Gram- negative SCL benchmarks. Finally, the highest results are achieved by the consensus of the individual predictions based on PSSM profiles and GO terms. To provide more information about the statistical significance of our proposed predictive model achieved results, we examined the confusion matrix which gives statistics by subcellular location as it is shown in Table 5 for Gram-negative bacteria and Table 6 for Gram-positive bacteria, respectively. The results show that the overall performance of the combined predictions of each individual model is significantly improved, accordingly, the consensus decision of individual models decisions based on different features works better than features fusion in discriminating between the different compartments. The results support our assumption that combining the decisions of diverse individual prediction models can significantly enhance the performance of SCL prediction. Moreover, the incorporated features are biologically more meaningful and need further attention to be well analyzed and exploited in proteins related problems.

**Table 5:** Performance evaluation by 5-fold cross-validation tests on Gram-negative bacteria proteins using a consensus of PSSM and GO terms-based predictions. The multi-label confusion matrix reflects well the predictions performance for each location separetely.

Subcellular locations		Metrics										
		TP	FP	FN	TN	Correct	Wrong	% TP	% FP	% FN	% TN	% Correct
4,152	Cytoplasm (C)	4,132	157	20	2,269	6,401	177	0.63	0.02	0	0.34	0.97
1,415	Inner membrane (I)	1,388	238	27	4,925	6,313	265	0.21	0.04	0	0.75	0.96
346	Outer membrane (O)	315	29	31	6,203	6,518	60	0.05	0	0	0.94	0.99
422	Periplasm (P)	393	72	29	6,084	6,477	101	0.06	0.01	0	0.92	0.98
272	Extracellular (S)	250	39	22	6,267	6,517	61	0.04	0.01	0	0.95	0.99
10	Vacuole (V)	8	0	2	6,568	6,576	2	0	0	0	1	1

**Table 6:** Performance evaluation by 5-fold cross-validation tests on Gram-positive bacteria proteins using a consensus of both GO terms and PSSM profiles predictions. The multi-label confusion matrix reflects well the predictions performance for each location separetely.

Subcellular locations		Metrics										
		TP	FP	FN	TN	Correct	Wrong	% TP	% FP	% FN	% TN	% Correct
349	Cytoplasm (C)	323	30	26	2,069	2,392	56	0.13	0.01	0.01	0.85	0.98
1,779	Inner membrane (I)	1,768	61	11	608	2,376	72	0.72	0.02	0	0.25	0.97
290	Extracellular (S)	282	101	8	2,057	2,339	109	0.12	0.04	0	0.84	0.96
34	Cell wall (W)	13	3	21	2,411	2,424	24	0.01	0	0.01	0.98	0.99
4	Vacuole (V)	4	0	0	2444	2448	0	0	0	0	1	1

### 3.4 Multiple location prediction

**Figure 8:**
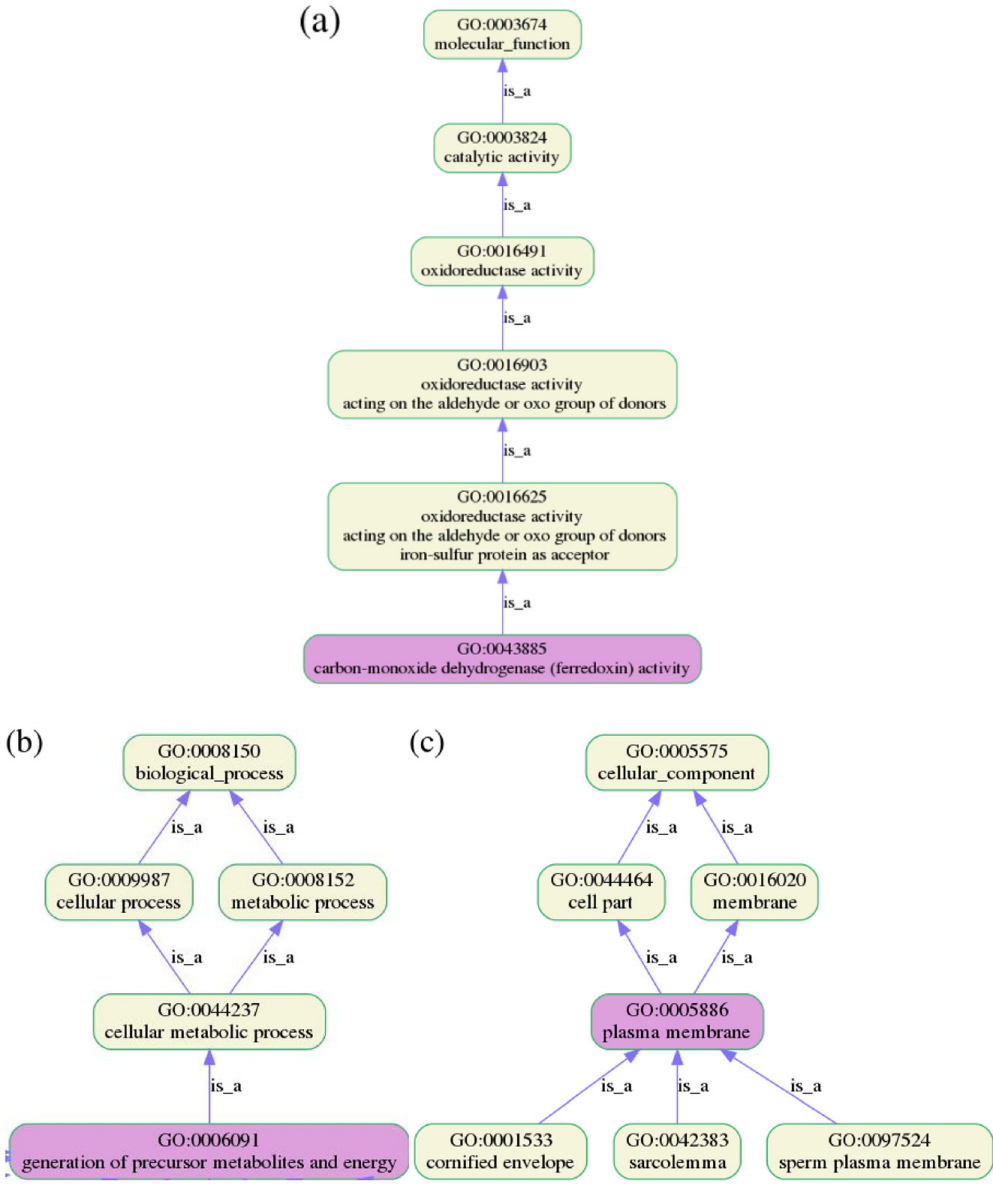
Effect of the presence of the MF GO term GO:0043885, the BP GO term GO:0006091, and the CC GO term GO:0005886 on the proposed model prediction of COOS2_CARHZ (C/I) and COOS1_CARHZ(C/I) proteins where it really succeeded, while they are predicted as cytoplasm (C) or membrane (M) by the others predictors.

There are only eight multiple sites proteins in Gram-positive bacterial dataset with the statistics I/S (1), C/S (2), S/W (2), and I/C (3) as it is shown in Figure 5. Gram-negative bacterial dataset contains 39 multiple sites proteins among them the minority mutli-label classes are C/O (1), C/S (2), I/O (2), I/P (2), O/S (4), and P/S (4); whereas I/C (18) and C/P (6) are more populated as is highlighted in Figure 4. We have thus, compared the predictions provided by our consensus model against three state-of-art SCL prediction methods, namely CELLO2GO24
https://cello.life.nctu.edu.tw/cello2go/. [49], BUSCA25
https://busca.biocomp.unibo.it/. [32] and UniLoc26
https://bioapp.iis.sinica.edu.tw/UniLoc/. [121]. CELL2GO is based on GO terms and uses BLAST to search for homologous sequences. The recent method BUSCA combines three SCL prediction methods (BaCelLo [41], MemLoci [122] and SChloro [123]) and methods for identifying signal and transit peptides, glycophosphatidylinositol (GPI)-anchors and transmembrane domains. Whereas UniLoc SCL prediction is based on the implicit similarity between proteins, it identifies template proteins based on the number of shared related words using PSI-BLAST search against NCBI nr27
ftp://ftp.ncbi.nlm.nih.gov/blast/db/. database. The results reported in Table 7 and Table 8 show how the predicted locations have been obtained by the consensus model for the minority multi-label classes in Gram-positive and Gram-negative bacterial proteins datasets respectively. One should be careful in drawing conclusions when comparing SCL methods, since they differ in many aspects such as the learning strategy, features that they learn from since they are extracted from different sources of information, and their coverage of different localizations which depends on the available classes in the learning datasets [124]. In addition each SCL prediction method has its strengths and disadvantages and an objective comparison is practically difficult. Here we focused on the minority multi-label classes in both Gram-positive and Gram-negative bacterial benchmarks to observe how they are discerned by the different predictors. It appears that the number of essential GO terms from the MF and BP categories is significantly larger than that from the CC category for both Gram-negative and Gram-positive bacterial datasets. From Table 7, we observe that our proposed model predicts membrane proteins (assigned to M class by the others) as either cytoplasmic or inner membrane or both which is somehow a good result as shown by Figure 8 obtained using GOATOOLS28
https://github.com/tanghaibao/goatools. software [125].

**Table 7:** The proposed SCL prediction model predictions for multiple sites proteins of Gram-positive bacterial dataset versus CELLO2GO, BUSCA, and UniLoc predictions.

Protein name True location (s)	Predicted essential GO terms			Top ranked CC description	Predicted location (s)						
	MF	BP	CC		PseAAC	PSSM profiles	GO terms	Consensus	CELLO2GO	BUSCA	UniLoc
COOS2_CARHZ C/I	GO:0043885 GO:0018492 GO:0016151 GO:0051539 GO:0051538 GO:0008198 GO:0042803 GO:0016746	GO:0006091 GO:0055114 GO:0006084 GO:0015977	GO:0005886 GO:0005737 GO:0032991	plasma membrane cytoplasm protein-containing complex	I	I	C/I	C/I	C	M	C/M
COMGG_BACSU I/S		GO:0030420	GO:0016021 GO:0005576 GO:0005886	integral component of membrane extracellular region plasma membrane	I	C	C	C	S/M	M	M/S
COOS1_CARHZ C/I	GO:0043885 GO:0018492 GO:0016151 GO:0051539 GO:0051538 GO:0008198 GO:0042803 GO:0050418 GO:0004601	GO:0006091 GO:0055114 GO:0042542 GO:0098869 GO:0006807	GO:0005886 GO:0005737 GO:0032991	plasma membrane cytoplasm protein-containing complex	I	I	I	I	C	M	C/M
ECPA_STAEP S/W	GO:0008234	GO:0006508 GO:0009405	GO:0005576 GO:0005618	extracellular region cell wall	S	S	S	S	S	C	S/W
LYS_CLOAB C/S	GO:0003796 GO:0008233 GO:0008745	GO:0016998 GO:0009253 GO:0042742 GO:0019835 GO:0006508	GO:0005576 GO:0005737 0016021	extracellular region cytoplasm integral component of membrane	S	S	S	S	S	C	S
HYES_CORS2 C/I	GO:0016787 GO:0016746 GO:0140096	GO:0019439 GO:0006508	GO:0005886 GO:0005737	plasma membrane cytoplasm	I	I	C	C/I	M/C	C	M
CPLR_DESHA S/W	GO:0050781 GO:0031419 GO:0016787 GO:0000166 GO:0061783 GO:0046872 GO:0003723 GO:0016301	GO:0046193 GO:0009166 GO:0055114 GO:0016998 GO:0009253 GO:0042742 GO:0019835 GO:0016311 GO:0015031 GO:0016310	GO:0009275 GO:0005576 GO:0005886 GO:0016021	cell wall extracellular region plasma membrane integral component of membrane	I	S	C	C/S	S	S	S/M/W
PLC_LISMO C/S	GO:0008081 GO:0004436	GO:0006629 GO:0009405 GO:1901575 GO:0006508	GO:0005576 GO:0005737 GO:0016021	extracellular region cytoplasm integral component of membrane	I	S	S	S	S	M	S

**Table 8:** The proposed SCL prediction model predictions for some multiple sites proteins of Gram-negative bacterial dataset versus CELLO2GO, BUSCA, and UniLoc predictions.

Protein name	Predicted essential GO terms			Top ranked CC description	Predicted location (s)						
True location (s)	MF	BP	CC		PseAAC	PSSM profiles	GO terms	Consensus	CELLO2GO	BUSCA	UniLoc
CH60_NEIGO C/O	GO:0051082 GO:0005524	GO:0042026 GO:0006458	GO:0005737 GO:0101031 GO:0009279	cytoplasm chaperone complex cell outer membrane	C	C	C	C	C	C	C
ENO_ECOLI C/S	GO:0004634 GO:0000287 GO:0003883 GO:0042802 GO:0005524	GO:0006096 GO:0044210 GO:0006541	GO:0000015 GO:0009986 GO:0005576 GO:0005856 GO:0016020	phosphopyruvate hydratase complex cell surface extracellular region cytoskeleton membraneane	C	C	C	C	C	C	C/S
SPIC_SALTY C/S		GO:0009405 GO:0015031 GO:0035592 GO:0002790 GO:0032940	GO:0005576 GO:0009279 GO:0005737	extracellular region cell outer membrane cytoplasm	C	I	S	I/S	S	C	C/S
MXIG_SHIFL I/O		GO:0009405	GO:0016021 GO:0009279 GO:0005886	extracellular region cell wall	C	I	I	I	I/C	C	M
PGPB_ECOLI I/O	GO:0008962 GO:0000810 GO:0008195 GO:0050380	GO:0016311 GO:0009395 GO:0006655 GO:0009252	GO:0016021 GO:0009279 GO:0005886	integral component of membrane cell outer membrane plasma membrane	I	I	I	I	I	M	M
LEPA_ECOLI I/P	GO:0043022 GO:0003746 GO:0003924 GO:0005525 GO:0043024 GO:0043023 GO:0042802 GO:0016779	GO:0045727 GO:0006414 GO:0009651 GO:0009268 GO:0009409	GO:0005886 GO:0005829	integral component of membrane cell outer membrane plasma membrane	C	C	C	C	I/C	C	C/M
LEPA_SALTY I/P	GO:0043022 GO:0003746 GO:0003924 GO:0005525	GO:0045727 0006414	GO:0005886	plasma membrane cytosol	I	S	C	C/S	I/C	C	C/M
TIBA_ECOLI O/S	GO:0005509 GO:0004674 GO:0005524	GO:0009405 GO:0007155 GO:0006468	GO:0019867 GO:0009986 GO:0044462 GO:0030313 GO:0042597 GO:0005576 GO:0016021	outer membrane cell surface external encapsulating structure part cell envelope periplasmic space extracellular region	S	S	S	S	S/O	C	M
FRPC_NEIMC O/S	GO:0005509 GO:0090729 GO:0004035 GO:0008780 GO:0004553	GO:0009405 GO:0016311 GO:0005975 GO:0007156	GO:0005576 GO:0009279 GO:0016021	extracellular region cell outer membrane integral component of membrane	O	S	S	S	S/C	C	S/C
YOPM_YERPE O/S	GO:0004842 GO:0016874 GO:0043531 GO:0004672 GO:0016787 GO:0005524	GO:0016567 GO:0007154 GO:0023052 GO:0051716 GO:0050794 GO:0043326	GO:0045335 GO:0016021	phagocytic vesicle integral component of membrane	C	C	C	C	C	S/I/C	S/C
FRPA_NEIMC O/S	GO:0005509 GO:0004035 GO:0004190	GO:0009405 GO:0016311 GO:0006629 GO:0006508	GO:0005576 GO:0016020	extracellular region membrane	O	S	S	S	S	C	M/S
TCPS_VIBCH P/S		GO:0009297 GO:0009405	GO:0042597 GO:0005576	periplasmic space extracellular region	C	I	S	I/S	P/C	S	P/S
PPBL_PSEAE S/P	GO:0042301 GO:0004035	GO:0035435 GO:0016311 GO:0015628 GO:0043952	GO:0043190 GO:0042597 GO:0005576	ATP-binding cassette (ABC) transporter complex periplasmic space extracellular region	S	S	P	S/P	S	S	S/P/M
HLYE_ECOLI S/P	GO:0090729 GO:0042802	GO:0044179 GO:0009405 GO:0044532	GO:0020002 GO:0042597 GO:0005576 GO:0016021	host cell plasma membrane periplasmic space extracellular region integral component of membrane	C	C	S	S/C	S	C	P/M/S
PPBH_PSEAE S/P	GO:0090729 GO:0042802	GO:0016311 GO:0015628 GO:0043952	GO:0042597 GO:0005576	periplasmic space extracellular region	C	P	P	P	P	S	P/S

We also observe that the other proteins are reasonably predicted and that in the particular case of a total absence of GO terms, which means that even when the prediction is left to chance (arbitrary prediction), the proposed prediction model remains robust. From Table 7 and Table 8, we can show that our prediction model recognizes plasma membrane as either inner membrane or cytoplasm and has a strong ability to recognize both cytoplasm and extracellular space classes. Periplasmic space is also well recognized whereas the main difficulty remains in cell wall class prediction. The results show that some GO terms have a critical influence on the decision even in the presence of the others GO terms. For example, the pathogenesis BP GO term GO:0009405 is a good indicator of inner membrane as well as the CC GO term GO:0016021. The BP GO term GO:0016311 reflects the periplasm class whereas the presence of the MF GO term GO:0005509 induces extracellular space. However, it is noticed that even in the absence of MF GO terms, the prediction remains correct, which suggests that the three types of GO terms are complementary for the SCL prediction and when all the three GO categories are present, the prediction is more effective. The reported results show that the decisions of the compared models are roughly complementary and seemingly, taking into account all their decisions might enhance the prediction quality. Further results concerning the more populated multi-label classes I/C (18) and C/P (6) are reported in Supplementary material S2. This experimental evaluation has shown the effectiveness of the proposed multi-label prediction model. However, more investigations are necessary to learn more about GO terms influence on the prediction model decision. We believe that the proposed model effectiveness would be more significant when the benchmark datasets contain more training samples and cover more subcellular location sites. Finally, in order to satisfy the Chou’s fifth rule in our future work, we shall provide a web-server for the method presented in this paper. The related datasets can be download from: https://github.com/hb-sources/Protein-SCL-Prediction and the source code for implementing in this study is available from the author upon request.

## 4 Conclusion

Protein SCL prediction is a challenging problem by its nature since it is an imbalanced multi-label classification problem. Imbalanced because most of the training datasets have an uneven distribution of the proteins in different organelles and muti-label, due to the inherent ability of proteins to simultaneously reside at, or move between two or more different subcellular location sites. In this study, we have proposed an ensemble multi-label SCL prediction system that exploits the potential discriminative power of evolutionary information in the form of PSSM profiles and GO terms to tackle Gram-positive and Gram-negative SCL prediction problem. We have shown that combining individual prediction models decisions is better than features fusion and the assumption that better performance could be expected by combining uncorrelated output predictions since each individual model performs differently proved to be more realistic. Moreover, the results show the superiority of evolutionary information-based prediction, especially when GO annotation is considered, which highlights the usefulness of sequence and structure homology for inferring protein localization and improving the prediction correctness. In the proposed prediction model we have exploited the correlations embedded in label space by using label powerset (LP) transformation strategy with in mind a flat organization structure of the SCLs. However, since proteins trafficking in the cell is highly correlated to the subcellular location sites relationships and organization, in our following research attempts will be made to implement a multiple prediction system considering interdependences between subcellular locations. Investigating the effect of an hierarchical structure organization of the location sites might be an interesting avenue in order to obtain a more effective prediction. Further work will include efforts on collecting more annotated proteins to learn from larger datasets, to extend the coverage scope to further subcellular locations at least for Gram-negative bacteria such as fimbrium, flagellum and nucleoid, and to extend the approach to other organisms. However, further investigations are required to shed light on the underlying mechanisms that govern the positioning of proteins in specific cell sites and on how they are implicated in human diseases.
