# Supplementary material for: Use of Chou’s 5-steps rule to predict the subcellular localization of gram-negative and gram-positive bacterial proteins by multi-label learning based on gene ontology annotation and profile alignment
Source: J Integr Bioinform. 2020 Jun 29;18(1):51–79. doi: 10.1515/jib-2019-0091 (PMC8035964; doi:10.1515/jib-2019-0091)
Supplement: Supplementary file 1 [file jib-18-20190091-s001.pdf]

Use of Chou's 5-Steps Rule to Predict the Subcellular Localization of Gram-Negative and Gram-Positive Bacterial Proteins by Multi-Label Learning based on Gene Ontology Annotation and Profile Alignment

Hafida Bouziane and Abdallah Chouarfia
Département d'Informatique
Université des Sciences et de la Technologie d'Oran Mohamed Boudiaf, USTO-MB
BP 1505, El M'Naouer, 31000 Oran Algérie
e-mail: (hafida.bouziane,chouarfia)@univ-usto.dz

Random Forest (RF) [1] has been evaluated against Support Vector Machine [2, 3] as base classifier for Label Powerset (LP) transformation to apply multi-label learning to predict subcellular localisation of Gram-negative bacterial and Gram-positive bacterial proteins. The R package randomForest[1] has been used with different nbtree parameter settings and the software LIBSVM[2] [4] has been employed with a radial basis function (RBF) as the kernel function. The regularization parameter C and the kernel width parameter γ were optimized using grid search approach on the training set by 5-fold cross validation. The results reported in both Fig.1 and Fig.2 show that SVM outperformed RF only in the case when it learned from PseACC protein features, while RF was the best model when using PSSM profiles and GO terms as feature vectors.

References

[1] L. Breiman, "Random Forests," *Machine Learning*, vol. 45, no. 1, pp. 5–32, 2001. 1

[2] C. Cortes and V. Vapnik, "Support-vector networks," *Machine Learning*, vol. 20, no. 3, pp. 273–297, 1995. 1

[3] V. Vapnik, *Statistical Learning Theory*. John Wiley & Sons, Inc., New York, 1998. 1

[4] C. Chang and C. Lin, "LIBSVM : a library for support vector machines," *SIAM J. Appl. Math*, 2001. Software available at http://www.csie.ntu.edu.tw/ cjlin/libsvm. 1

[1] https://cran.r-project.org/web/packages/randomForest/index.html
[2] http://www.csie.ntu.edu.tw/~cjlin/libsvm

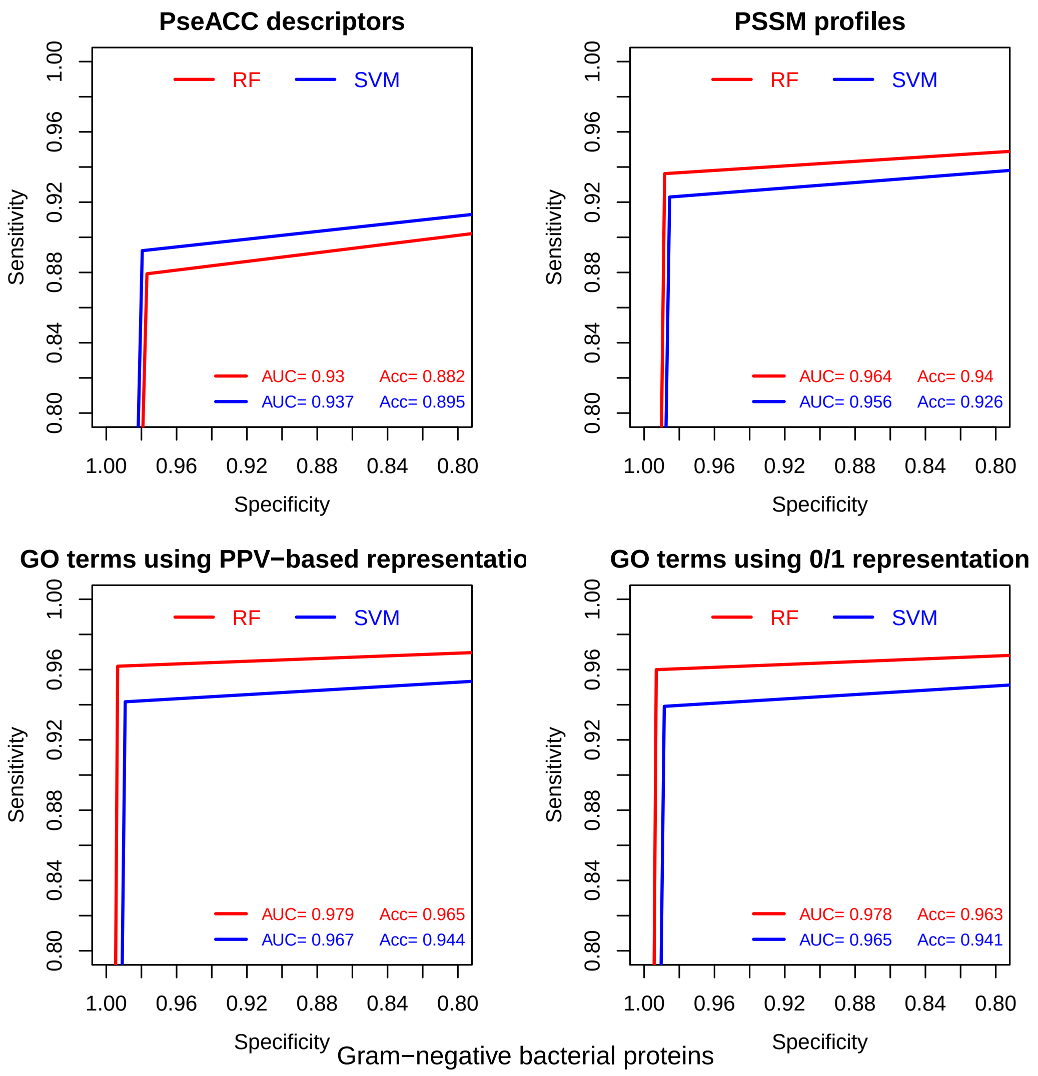

Fig. 1: Random Forest (RF) versus Support Vector Machine (SVM) as base classifier for Gram-negative bacterial proteins subcellular localization prediction.

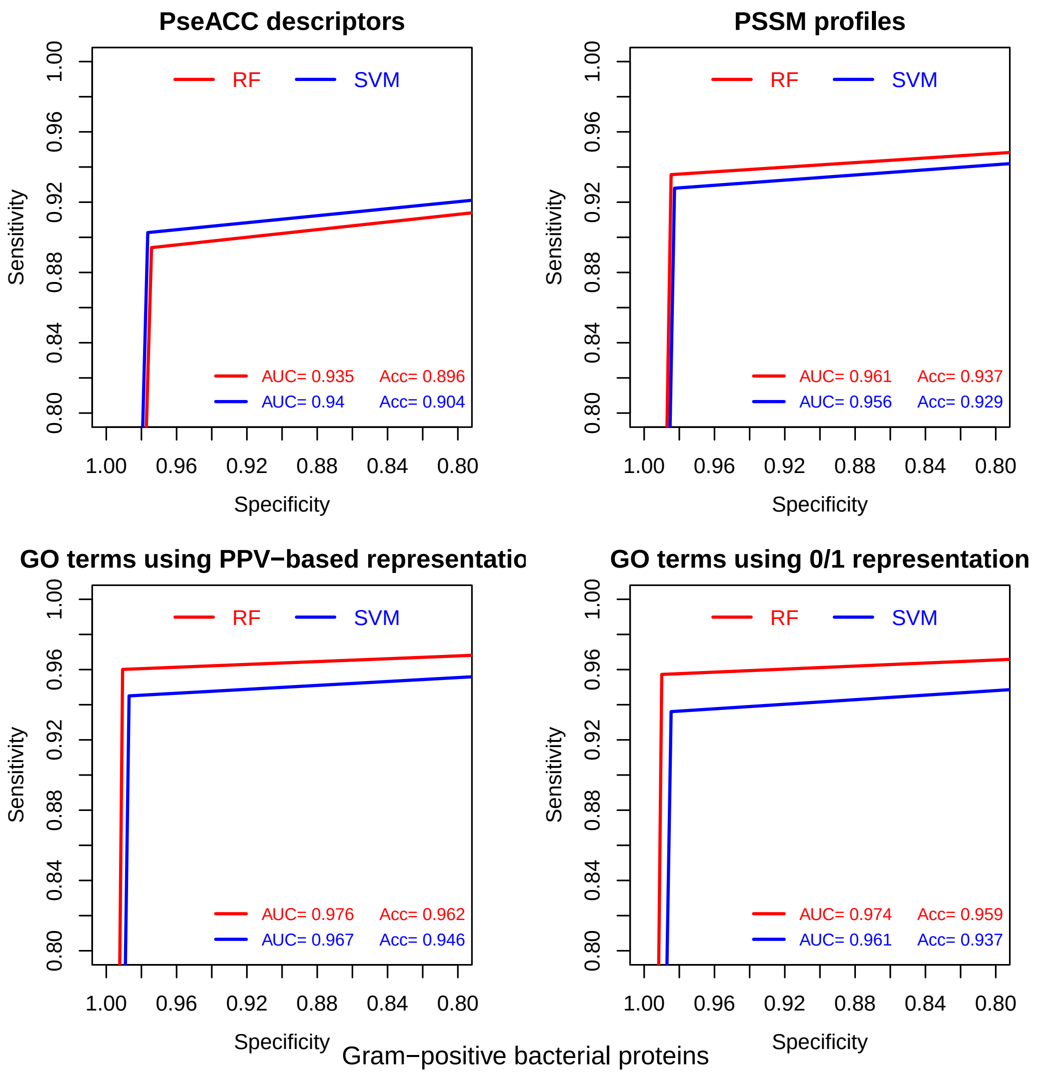

Fig. 2: Random Forest (RF) versus Support Vector Machine (SVM) as base classifier for Gram-positive bacterial proteins subcellular localization prediction.
