# Supplementary material for: Use of Chou’s 5-steps rule to predict the subcellular localization of gram-negative and gram-positive bacterial proteins by multi-label learning based on gene ontology annotation and profile alignment
Source: J Integr Bioinform. 2020 Jun 29;18(1):51–79. doi: 10.1515/jib-2019-0091 (PMC8035964; doi:10.1515/jib-2019-0091)
Supplement: Supplementary file 2 [file jib-18-20190091-s002.pdf]

Use of Chou's 5-Steps Rule to Predict the Subcellular Localization of Gram-Negative and Gram-Positive Bacterial Proteins by Multi-Label Learning based on Gene Ontology Annotation and Profile Alignment

Hafida Bouziane and Abdallah Chouarfia
Département d'Informatique
Université des Sciences et de la Technologie d'Oran Mohamed Boudiaf, USTO-MB
BP 1505, El M'Naouer, 31000 Oran Algérie
e-mail: (hafida.bouziane,chouarfia)@univ-usto.dz

The results reported in Tab.1, Tab.2 and Tab.3 show how the predicted locations have been obtained by the consensus model for the remaining multi-sites proteins in Gram-negative bacterial proteins dataset, where 18 proteins are localized in cytoplasm and inner membrane regions (I/C) and 6 proteins in cytoplasm and periplasm regions (C/P). The predictions provided by our consensus model have been compared against three state-of-art SCL prediction methods, namely CELLO2GO[1] [1], BUSCA[2] [2] and UniLoc[3] [3]. CELL2GO is based on GO terms and uses BLAST to search for homologous sequences. The recent method BUSCA combines three SCL prediction methods (BaCelLo [4], MemLoci [5] and SChloro [6]) and methods for identifying signal and transit peptides, glycophosphatidylinositol (GPI)-anchors and transmembrane domains. Whereas UniLoc SCL prediction is based on the implicit similarity between proteins, it identifies template proteins based on the number of shared related words using PSI-BLAST search against NCBI nr[4] database.

References

[1] C. Yu, C. Cheng, W. Su, K. Chang, S. Huang, J. Hwang, and C. Lu, "CELLO2GO:A Web Server for Protein subCELlular LOcalization Prediction with Functional Gene Ontology Annotation," *PLoS ONE*, vol. 9, no. 6, p. e99368, 2014. 1

[2] C. Savojardo, P. Martelli, P. Fariselli, G. Profiti, and R. Casadio, "BUSCA: an integrative web server to predict subcellular localization of proteins," *Nucleic Acids Research*, vol. 46, no. W1, pp. W459–W466, 2018. 1

[3] H. Lin, C. Chen, T. Sung, and W. Hsu, "UniLoc: A universal protein localization site predictor for eukaryotes and prokaryotes," *bioRxiv*, 2018. 1

[1] http://cello.life.nctu.edu.tw/cello2go/
[2] http://busca.biocomp.unibo.it/
[3] http://bioapp.iis.sinica.edu.tw/UniLoc/
[4] ftp://ftp.ncbi.nlm.nih.gov/blast/db/

Protein name	Predicted essential GO terms			Top ranked CC description	Predicted location (s)						
True location (s)	MF	BP	CC		PseAAC	PSSM profiles	GO terms	Consensus	CELLO2GO	BUSCA	UniLoc
RNI_AERHY<br>C/P	GO:0033897<br>GO:0003723<br>GO:0008847	GO:0090502	GO:0042597<br>GO:0005737	periplasmic space<br>cytoplasm	C	I	P	I/P	P	C	C/S
SECM_ECOLI<br>C/P	GO:0045182	GO:0006417	GO:0005829<br>GO:0042597	cytosol<br>periplasmic space	I	I	P	I/P	C/P	C	C/P
FKBB_ECOLI<br>C/P	GO:0003755<br>GO:0005528<br>GO:0042802	GO:0000413<br>GO:0006457	GO:0042597<br>GO:0005829	periplasmic space<br>cytosol	C	O	P	P/O	C	C	C/P
PLCR_PSEAE<br>C/P			GO:0042597<br>GO:0005737	periplasmic space<br>cytoplasm	C	I	P	I/P	S/P	C	P
PA1L_PSEAE<br>C/P	GO:0030246	GO:0007157	GO:0009986<br>GO:0042597<br>GO:0005737	cell surface<br>periplasmic space<br>cytoplasm	S	I	P	I/P	C/P	C	C/P
RNI_ECOLI<br>C/P	GO:0033897<br>GO:0008847<br>GO:0003723<br>GO:0016829	GO:0090502<br>GO:0006401	GO:0030288<br>GO:0005737	outer membrane-bounded periplasmic space<br>cytoplasm	P	P	P	P	P	S	C/P

Tab. 1: The proposed SCL prediction model predictions for some multiple sites proteins localized in cytoplasmic and periplasmic spaces of Gram-negative bacterial dataset versus CELLO2GO, BUSCA and UniLoc predictions.

Protein name	Predicted essential GO terms			Top ranked CC description	Predicted location (s)						
True location (s)	MF	BP	CC		PseAAC	PSSM profiles	GO terms	Consensus	CELLO2GO	BUSCA	UniLoc
IUCD_ECOLI /I	GO:0047091<br>GO:0008233	GO:0055114<br>GO:0046442<br>GO:0072351<br>GO:0055072<br>GO:0042398<br>GO:0051188<br>GO:0006508	GO:0005886<br>GO:0005737	plasma membrane<br>cytoplasm	C	C	I	C/I	C/I	C/M	C/M
KAIB_SYNP7<br>C/I	GO:0005515	GO:0007623<br>GO:0042326<br>GO:0009649	GO:0005886<br>GO:0005737	plasma membrane<br>cytoplasm	C	C	C	C	C	C	C/M
PNP_ECOLI<br>C/I	GO:0004654<br>GO:0000287<br>GO:0003723<br>GO:0000175<br>GO:0035438<br>GO:0042802<br>GO:0016301	GO:0006402<br>GO:0006396<br>GO:0009408<br>GO:0090503<br>GO:0016310	GO:0005737<br>GO:0016020	cytoplasm<br>membrane	C	C	C	C	C	C	C
PTNAB_ECOLI<br>C/I	GO:0008982<br>GO:0103111<br>GO:0015578<br>GO:0016301<br>GO:0005515	GO:0034219<br>GO:0009401<br>GO:0016310	GO:0005737<br>GO:0016021<br>GO:0005886	cytoplasm<br>integral component of membrane<br>plasma membrane	C	C	I	C/I	C	C	C
YSCB_YERPE<br>C/I	GO:0005515	GO:0050708	GO:0005737<br>GO:0005886	cytoplasm<br>plasma membrane	I	I	I	I	C	C	C/M
DMSD_ECOLI<br>C/I	GO:0005048<br>GO:0005515	GO:0061077	GO:0031234<br>GO:0005737	extrinsic component of cytoplasmic side of plasma membrane<br>cytoplasm	C	C	C	C	I	C	C/M
DLDH_ECOLI<br>C/I	GO:0004148<br>GO:0050660<br>GO:0009055<br>GO:0042802<br>GO:0015036<br>GO:0008270	GO:0045454<br>GO:0022900<br>GO:0006096<br>GO:0006103<br>GO:0019464<br>GO:0006979	GO:0005623<br>GO:0016020	cell<br>membrane	C	C	C	C	C	C	C
YHBG_ECOLI<br>C/I	GO:0016887<br>GO:0005524<br>GO:0015437<br>GO:0044877	GO:0055085<br>GO:0015920	GO:0043190<br>GO:0005737	ATP-binding cassette (ABC) transporter complex<br>cytoplasm	C	I	I	I	C/I	C	C/M
PSS_ECOLI<br>C/I	GO:0017169<br>GO:0016787	GO:0032049	GO:0005829<br>GO:0005886	cytosol<br>plasma membrane	C	C	I	C/I	C/I	C	C/M

Tab. 2: The proposed SCL prediction model predictions for some multiple sites proteins localized in cytoplasmic and inner membrane regions of Gram-negative bacterial dataset versus CELLO2GO, BUSCA and UniLoc predictions.

[4] A. Pierleoni, P. Martelli, P. Fariselli, and R. Casadio, "BaCelLo: a balanced subcellular localization predictor," *Bioinformatics*, vol. 22, no. 14, pp. e408–e416, 2006. 1

[5] A. Pierleoni, P. Martelli, and R. Casadio, "MemLoci: predicting subcellular localization of membrane proteins in eukaryotes," *Bioinfor-*

Protein name	Predicted essential GO terms			Top ranked CC description	Predicted location (s)						
True location (s)	MF	BP	CC		PseAAC	PSSM profiles	GO terms	Consensus	CELLO2GO	BUSCA	UniLoc
GSPA_AERHY<br>C/I	GO:0005524<br>GO:0008233<br>GO:0017111	GO:0051301<br>GO:0006508	GO:0016021<br>GO:0005886<br>GO:0005737	integral component of membrane<br>plasma membrane<br>cytoplasm	C	C	I	C/I	C/I	C	C/M
DNAK_ECOLI<br>C/I	GO:0051082<br>GO:0005524<br>GO:0051087<br>GO:0044183<br>GO:0016989<br>GO:0019904<br>GO:0043531<br>GO:0008270	GO:0006457<br>GO:0034620<br>GO:0009408<br>GO:0032984<br>GO:1903507<br>GO:0065003<br>GO:0006260	GO:0005829<br>GO:0005886<br>GO:0032991	cytosol<br>plasma membrane<br>protein-containing complex	C	C	I	C/I	C	C	C/M
HTPG_ECOLI<br>C/I	GO:0051082<br>GO:0005524<br>GO:0016301<br>GO:0042802<br>GO:0042623	GO:0006457<br>GO:0016310<br>GO:0009408<br>GO:0006974	GO:0005737<br>GO:0005886	cytoplasm<br>plasma membrane	C	C	C	C	C	C	C
NUOG_ECOLI<br>C/I	GO:0043546<br>GO:0048038<br>GO:0051537<br>GO:0008137<br>GO:0051539<br>GO:0009055<br>GO:0046872<br>GO:0005515	GO:0042773<br>GO:0009060	GO:0016020<br>GO:1990204<br>GO:0071944<br>GO:0005737	membrane<br>oxidoreductase complex<br>cell periphery<br>cytoplasm	C	C	I	C/I	C/I	C	M
NUOCD_ECOLI<br>C/I	GO:0048038<br>GO:0050136<br>GO:0051287<br>GO:0005515	GO:0055114	GO:0030964<br>GO:0005886<br>GO:0005737<br>GO:0098803<br>GO:1990204	NADH dehydrogenase complex<br>plasma membrane<br>cytoplasm<br>respiratory chain complex<br>oxidoreductase complex	C	C	I	C/I	C	C	C/M
FHUF_ECOLI<br>C/I	GO:0051537<br>GO:0016491<br>GO:0046872	GO:0055114<br>GO:0033212	GO:0005829<br>GO:0005886<br>GO:0016021	cytosol<br>plasma membrane<br>integral component of membrane	C	C	I	C/I	C/I	C	C/M
TRME_ECOLI<br>C/I	GO:0003924<br>GO:0005525<br>GO:0046872<br>GO:0019003<br>GO:0042802	GO:0006400<br>GO:0009268<br>GO:0061077<br>GO:0001510	GO:0005737<br>GO:0005886	cytoplasm<br>plasma membrane	C	C	C	C	C	C	C
SYCN_YERPE<br>C/I		GO:0009306	GO:0005886<br>GO:0005737	plasma membrane<br>cytoplasm	I	C	I	C/I	C/I	C	C/M

Tab. 3: The proposed SCL prediction model predictions for some multiple sites proteins localized in cytoplasmic and inner membrane regions of Gram-negative bacterial dataset versus CELLO2GO, BUSCA and UniLoc predictions.

matics, vol. 27, no. 9, pp. 1224–1230, 2011. 1

[6] C. Savojardo, P. Martelli, P. Fariselli, and R. Casadio, “SChloro: directing viridiplantae proteins to six chloroplastic sub-compartments,” *Bioinformatics*, vol. 33, no. 3, pp. 347–353, 2016. 1
